# White Pine Wood Bullet Probe

**Published:** 1867-02

**Authors:** 


*White Pine Wood Bullet Probe.*—Dr. V. Gelisch, of Los Angeles, Cal., recommends (*Pacific Med. and Surg. Journal*, Oct. 1866) a piece of white pine cut into the shape of a probe as a ready means of detecting a leaden ball in a wound. He says if such a probe be inserted into a wound and rubbed against the suspected object, and then quickly withdrawn, if the object be a leaden ball, traces of the lead will be plainly perceptible on the end of the probe. This will be useful where a Nelaton's probe is not at hand.

